# Molecular pathology and synaptic loss in primary tauopathies: A [^18^F]AV-1451 and [^11^C]UCB-J PET study

**DOI:** 10.1093/brain/awab282

**Published:** 2022-03-29

**Authors:** Negin Holland, Maura Malpetti, Timothy Rittman, Elijah E Mak, Luca Passamonti, Sanne S Kaalund, Frank H. Hezemans, P. Simon Jones, George Savulich, Young T. Hong, Tim D. Fryer, Franklin I. Aigbirhio, John T O’Brien, James B Rowe

**Affiliations:** 1 Department of Clinical Neurosciences, University of Cambridge, Cambridge Biomedical Campus, Cambridge, CB2 0SZ; 2 Cambridge University Hospitals NHS Foundation Trust, Cambridge, UK, CB2 0QQ; 3 Department of Psychiatry, University of Cambridge, School of Clinical Medicine, Cambridge Biomedical Campus, UK, CB2 0QQ; 4 Istituto di Bioimmagini e Fisiologia Molecolare (IBFM), Consiglio Nazionale delle Ricerche (CNR), 20090, Milano, Italy; 5 Wolfson Brain Imaging Centre, University of Cambridge, UK, CB2 0QQ; 6 Medical Research Council Cognition and Brain Sciences Unit, University of Cambridge, UK, CB2 7EF

**Keywords:** Primary tauopathies, PSP, CBD/CBS, synapse, tau

## Abstract

The relationship between in vivo synaptic density and molecular pathology in primary tauopathies is key to understanding the impact of tauopathy on functional decline and in informing new early therapeutic strategies. In this cross-sectional observational study, we determine the in vivo relationship between synaptic density and molecular pathology, in the primary tauopathies of Progressive Supranuclear Palsy (PSP) and Corticobasal Degeneration (CBD), as a function of disease severity. Twenty three people with PSP, and twelve people with Corticobasal Syndrome (CBS) were recruited from a tertiary referral centre. Nineteen education, sex and gender-matched control participants were recruited from the National Institute for Health Research ‘Join Dementia Research’ platform. Cerebral synaptic density and molecular pathology, in all participants, were estimated using PET imaging with the radioligands [^11^C]UCB-J and [^18^F]AV-1451, respectively. Patients with CBS also underwent amyloid PET imaging with [^11^C]PiB to exclude those with likely Alzheimer’s pathology – we refer to the amyloid negative cohort as having CBD although acknowledge other pathologies exist. Disease severity was assessed with the PSP rating scale; regional non-displaceable binding potentials (BP_ND_) of [^11^C]UCB-J and [^18^F]AV-1451 were estimated in regions of interest from the Hammersmith Atlas, excluding those with known off-target binding for [^18^F]AV-1451. As an exploratory analysis, we also investigated the relationship between molecular pathology in cortical brain regions, and synaptic density in subcortical areas. Across brain regions, there was a positive correlation between [^11^C]UCB-J and [^18^F]AV-1451 BP_ND_ (β = 0.4, t = 3.6, p = 0.001), independent of age or time between PET scans. However, this correlation became less positive as a function of disease severity in patients (β = - 0.02, t = -2.9, p = 0.007, R = -0.41). Between regions, cortical [^18^F]AV-1451 binding was negatively correlated with synaptic density in subcortical areas (caudate nucleus, putamen). Brain regions with higher synaptic density are associated with a higher [^18^F]AV-1451 binding in PSP/CBD, but this association diminishes with disease severity. Moreover, higher cortical [^18^F]AV-1451 binding correlates with lower subcortical synaptic density. Longitudinal imaging is required to confirm the mediation of synaptic loss by molecular pathology. However, the effect of disease severity suggests a biphasic relationship between synaptic density and molecular pathology with synapse-rich regions vulnerable to accrual of pathological aggregates, followed by a loss of synapses in response to pathology. Given the importance of synaptic function for cognition, our study elucidates the pathophysiology of primary tauopathies and may inform the design of future clinical trials.

## Introduction

Synaptic loss is a feature of many neurodegenerative disorders ^1–3^. It is closely related to cognitive decline in symptomatic stages of disease ^4, 5^, but can begin long before symptom onset and neuronal loss ^6^. Synaptic loss and dysfunction may be an important mediator of decline even where atrophy is minimal or absent ^7, 8^. Conversely, synaptic connectivity may facilitate the spread of oligomeric mis-folded proteins such as tau ^9–14^. The relationship between synaptic loss and the accumulation of mis-folded proteins in primary tauopathies has yet to be determined *in vivo*. Preclinical models suggest early synaptotoxicity of oligomeric tau, leading to reduced synaptic plasticity and density ^15, 16^. In patients with mutations of microtubule-associated protein tau (MAPT), there are deficiencies in many synaptic pathways including GABA-mediated signalling and synaptic plasticity ^17^. The mechanisms of synapse loss following tau pathology include both direct and indirect pathways (reviewed in Spires-Jones and Hyman ^18^), however, the severity of synaptic toxicity in the related tauopathy of Alzheimer’s disease appears to be dependent on the stage of disease in preclinical models, and in patients post-mortem and *in vivo*. In animal models of Alzheimer’s disease, and at human post-mortem, there is differential expression of synaptic proteins in the early stages with increases in some proteins and reductions in others ^19, 20^. This may be an attempt to maintain cellular physiology in early disease, which fails as the disease progresses, leading to loss of synaptic function and synapse numbers in moderate and advanced disease. In clinical disorders, the *in vivo* pathologies of synaptic density and tau burden can be characterised by positron emission tomography (PET). Recent *in vivo* PET imaging in Alzheimer’s disease using [^11^C]UCB-J as a marker of synaptic density and [^18^F]AV-1451 or [^18^F]MK-6240 PET as markers of tau pathology have shown decreased temporal lobe synaptic density with increasing pathological burden ^21^, but with individual variability depending on the severity of cortical pathology ^22^. However, the pathology of Alzheimer’s disease is multifaceted with amyloid and tau aggregation, vascular changes and neuroinflammation ^23^.

In this study, we use Progressive Supranuclear Palsy - Richardson’s syndrome (PSP) ^24^ and Corticobasal Degeneration (CBD) ^25^ as models of human tauopathy, with relevance to other tau-mediated neurodegenerative disorders, and examine the *in vivo* relationship between synaptic density and burden of molecular pathology. An advantage of studying PSP is the very high correlation between the clinical syndrome, and the specific 4R-tauopathy at autopsy ^26, 27^. The clinical phenotype of Corticobasal Syndrome (CBS), may be caused by CBD, but can also be mimicked by Alzheimer’s disease, and less commonly by other forms of frontotemporal lobar degeneration. Here, we use the term CBD to refer to patients with CBS in whom Alzheimer’s disease is excluded by [^11^C]PiB PET, whereby in the absence of amyloid pathology there is a high clinicopathological correlation with 4R-tauopathy at post-mortem. Both PSP and CBD demonstrate synaptic loss *in vivo* ^7, 8^ and at post-mortem ^1, 2^. The distribution of tau pathology in both diseases is well characterised with cortical and subcortical involvement ^28, 29^. Animal models of tauopathy have illustrated the co-localisation of tau aggregates at the synaptic bouton, associated with synaptic dysfunction and synaptic loss ^18, 30^ but the tau-synapse association is yet to be determined *in vivo*. [^18^F]AV-1451 signals are above normal in the cortex of patients with PSP and CBS/CBD ^31–37^ but there is relatively low affinity for 4R-tauopathy compared to Alzheimer’s disease, and off-target binding particularly in the basal ganglia. We therefore refer to [^18^F]AV-1451 as a marker of ‘molecular pathology’, referring to the combination of tau and non-tau targets.

**Figure 1:**
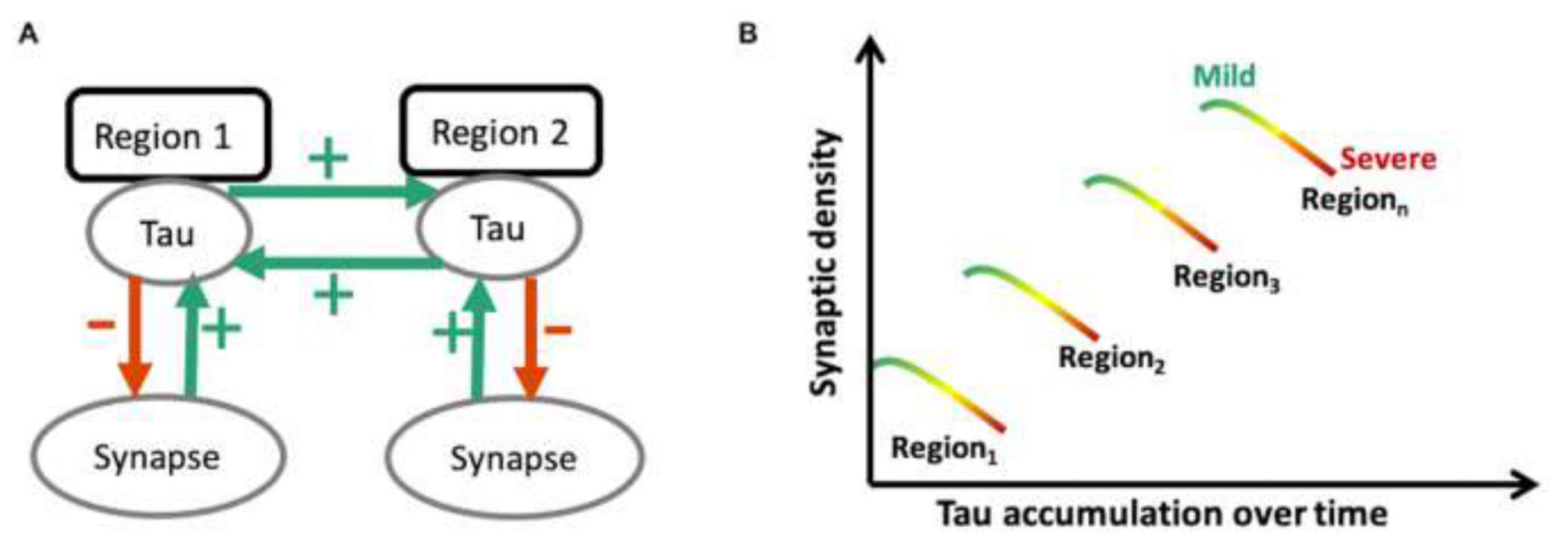
Schematic diagram illustrating the predicted toxic effect of tau on synaptic density as a function of disease severity. At a regional level (A) synaptic density promotes the spread of tau within the region, but also from one region to another functionally connected region (for example from Region 1 to Region 2 or vice versa; depicted by green arrows). Tau is however toxic to synapses, such that at a regional level it leads to a loss of synapses as the disease progresses. (B) Tau burden within a given region therefore, depends on a region’s baseline synaptic density: for example, Region 3, with a high baseline synaptic density, would accumulate more tau in the mild stages of disease (green); but as the disease progresses over time, to moderate and advanced stages (yellow and red, respectively), with increasing tau accumulation, tau induced synapto-toxicity occurs with a decline in the number of synapses within any given region. The prediction would therefore be that, whilst in mild disease the degree of tau accumulation is dependent on baseline synaptic density, as the disease progresses this relationship breaks down, moving towards a negative association between tau accumulation and synaptic density.

Figure 1 illustrates our hypotheses. Previous studies suggest that the strength of connectivity within a region and between brain regions can promote the spread of tau pathology, in humans as in preclinical models ^9–14^. Therefore, we hypothesised that brain areas with higher synaptic density would develop more tau pathology (schematically represented by green arrows in Figure 1A). We predicted that the spatial distribution of molecular pathology, as measured with the PET radioligand [^18^F]AV-1451, would be correlated with synaptic density, as measured with the PET radioligand [^11^C]UCB-J (which binds to the presynaptic vesicle glycoprotein SV2A that is ubiquitously expressed in brain synapses ^38, 39^). Since pathology in a region may impair efferent projections, a corollary hypothesis is that tau accumulation in one region (source region) leads to diaschisis characterised by reduced synaptic density in the areas to which it connects (target regions).

A second part of the model describes the consequence of the pathology, which is to reduce synaptic density (schematically represented by red arrows in Figure 1A). The predicted result is a positive relationship between [^18^F]AV-1451 binding and synaptic loss, negatively moderated by disease severity (Figure 1B).

## Materials and Methods

### Participant recruitment and study design

Twenty-three people with probable PSP–Richardson Syndrome, and twelve people with probable CBS in whom Alzheimer’s disease was excluded with [^11^C]PiB PET, were recruited from a regional specialist National Health Service clinic at the Cambridge University Centre for Parkinson-plus. We refer to our amyloid-negative CBS cohort as having CBD but acknowledge other pathologies are possible. Nineteen healthy volunteers were recruited from the UK National Institute for Health Research Join Dementia Research (JDR) register. Participants were screened using the inclusion/exclusion criteria set out in Holland *et* al. 2020^8^. Eligible participants underwent clinical and cognitive assessments (Table 1) including the revised Addenbrooke’s Cognitive Examination (ACE-R), the mini-mental state examination (MMSE), and the Institute of Cognitive Neurology (INECO) frontal screening; disease severity was measured with the PSP rating scale, and the Cortical Basal ganglia Functional Scale (CBFS) ^40^. Participants underwent 3T MRI, [^18^F]AV-1451 PET, and [^11^C]UCB-J PET. The research protocol was approved by the Cambridge Research Ethics Committee (reference 18/EE/0059) and the Administration of Radioactive Substances Advisory Committee. All participants provided written informed consent in accordance with the Declaration of Helsinki.

### PET data acquisition and kinetic analysis

#### [^11^C]UCB-J PET

The procedure for [^11^C]UCB-J synthesis, PET data acquisition, image reconstruction and kinetic analysis was the same as in Holland *et* al. 2020. In brief, dynamic PET data acquisition was performed on a GE SIGNA PET/MR (GE Healthcare, Waukesha, USA) for 90 minutes immediately after injection, with attenuation correction using a multi-subject atlas method ^41^ and improvements to the MRI brain coil component ^42^. Emission image series were aligned using SPM12 (www.fil.ion.ucl.ac.uk/spm/software/spm12/), and rigidly registered to the T1-weighted MRI acquired during PET data acquisition (TR = 3.6 msec, TE = 9.2 msec, 192 sagittal slices, in plane resolution 0.55 x 0.55 mm, interpolated to 1.0 x 1.0 mm; slice thickness 1.0 mm). The Hammersmith atlas (http://brain-development.org) with modified posterior fossa regions was spatially normalized to the T1-weighted MRI of each participant using Advanced Normalisation Tools (ANTs) software ^43^. Regional time-activity curves were extracted following the application of geometric transfer matrix (GTM) partial volume correction (PVC ^44^) to each dynamic PET image. Regions of interest (ROIs) were multiplied by a binary grey matter mask (>50% on the SPM12 grey matter probability map smoothed to PET spatial resolution), with the exception of the subcortical grey matter regions pallidum, substantia nigra, pons and medulla. To assess the impact of PVC, time-activity curves were also extracted from the same ROIs without the application of GTM PVC (discussed below as “without partial volume correction”).

To quantify SV2A density, [^11^C]UCB-J non-displaceable binding potential (BP_ND_) was determined using a basis function implementation of the simplified reference tissue model ^45^, with the reference tissue defined in the centrum semiovale ^46, 47^.

#### [^18^F]AV-1451 PET

As for [^11^C]UCB-J, PET data acquisition was performed on a GE SIGNA PET/MR for 90 minutes after [^18^F]AV-1451 injection, with attenuation correction as described above for [^11^C]UCB-J. Image processing was also as given above for [^11^C]UCB-J, except that [^18^F]AV-1451 BP_ND_ was determined using a different basis function implementation of the simplified reference tissue model ^48^ and the reference tissue was defined in inferior cerebellar grey matter using a 90% threshold on the grey matter probability map produced by SPM12 smoothed to PET resolution.

#### [^11^C]PiB PET

Amyloid imaging using Pittsburgh Compound B ([^11^C]PiB) followed the protocol given in Holland *et* al. 2020 ^8^. [^11^C]PiB cortical standardised uptake value ratio (SUVR; 50-70 minutes post injection) was calculated using the whole cerebellum reference tissue as per the Centiloid Project methodology ^49^. A negative amyloid status was characterised by a cortical [^11^C]PiB SUVR less than 1.21 obtained by converting the Centiloid cut-off of 19 to SUVR using the Centiloid-to-SUVR transformation in Jack *et* al. 2017 ^50^.

#### Statistical analyses

We compared demographic and clinical variables between the diagnostic groups using ANCOVA, and chi-square tests where appropriate. We used a linear mixed effects model to assess the overall relationship between [^18^F]AV-1451 and [^11^C]UCB-J BP_ND_, with age and scan interval as covariates. To adjust for normal levels of tracer uptake from off-target binding not present in the reference region (over and above the correction for non-specific binding in the reference region), we normalised the patient BP_ND_ data against controls by subtracting the regional mean BP_ND_ values in controls from the data of each patient, for each region, for each tracer. Furthermore, we removed regions with previously reported off-target binding of [^18^F]AV-1451 (basal ganglia, and substantia nigra ^51^). The linear mixed effect model therefore included, normalised [^11^C]UCB-J as the dependent variable, normalised [^18^F]AV-1451 as the independent variable, and age and scan interval as covariates of no interest. To investigate the effect of individual variability on the relationship between [^11^C]UCB-J and [^18^F]AV-1451 BP_ND_, we used a linear model with the slope of [^11^C]UCB-J BP_ND_ as a function of [^18^F]AV-1451 BP_ND_ for each individual (extracted from the previous linear mixed effect model) as the dependent variable, and the PSP rating scale (a measure of disease severity) as the independent variable, and age as a covariate of no interest. To explore the correlation between [^11^C]UCB-J and [^18^F]AV-1451 BP_ND_ between regions, we calculated a correlation matrix between cortical [^18^F]AV-1451 binding and synaptic density in cortical and subcortical regions.

Analyses were performed with and without GTM partial volume correction, yielding similar results; we focus on partial volume corrected BP_ND_ to limit the potential effect of atrophy on our ligand cross-correlation but present data without partial volume correction in the supplementary material. Statistical analyses were implemented in R (version 3.6.2).

#### Data Availability Statement

The data that support the findings of this study are available from the corresponding author, upon reasonable request for academic (non-commercial) purposes, subject to restrictions required to preserve participant confidentiality.

## Results

### Demographics

The patients (PSP and CBD) and control groups were similar in age, sex, education and injected activity of [^11^C]UCB-J and [^18^F]AV-1451 (Table 1). We observed typical cognitive profiles for people with PSP and CBD: impaired on verbal fluency, memory and visuospatial domains of the ACE-R and MMSE, and the INECO frontal screening tool.

### Relationship between [^11^C]UCB-J BP_ND_ and [^18^F]AV-1451 BP_ND_

Compared to controls, patients had significantly higher [^18^F]AV1451 binding in the caudate nucleus, pallidum, putamen, and substantia surviving correction for multiple comparison (p< 0.05, False Discovery Rate (FDR) corrected) (Supplementary Figure 1A, Supplementary Table 2). As previously reported in a smaller cohort ^7, 8^, patients had significantly lower [^11^C]UCB-J binding across all cortical and subcortical areas compared to controls, which survived FDR correction (Supplementary Figure 1B, Supplementary Table 4). Summary statistics for regional [^18^F]AV1451 and [^11^C]UCB-J binding potentials in patients and controls are shown in Supplementary Tables 1 and 3, respectively.

**Figure 2:**
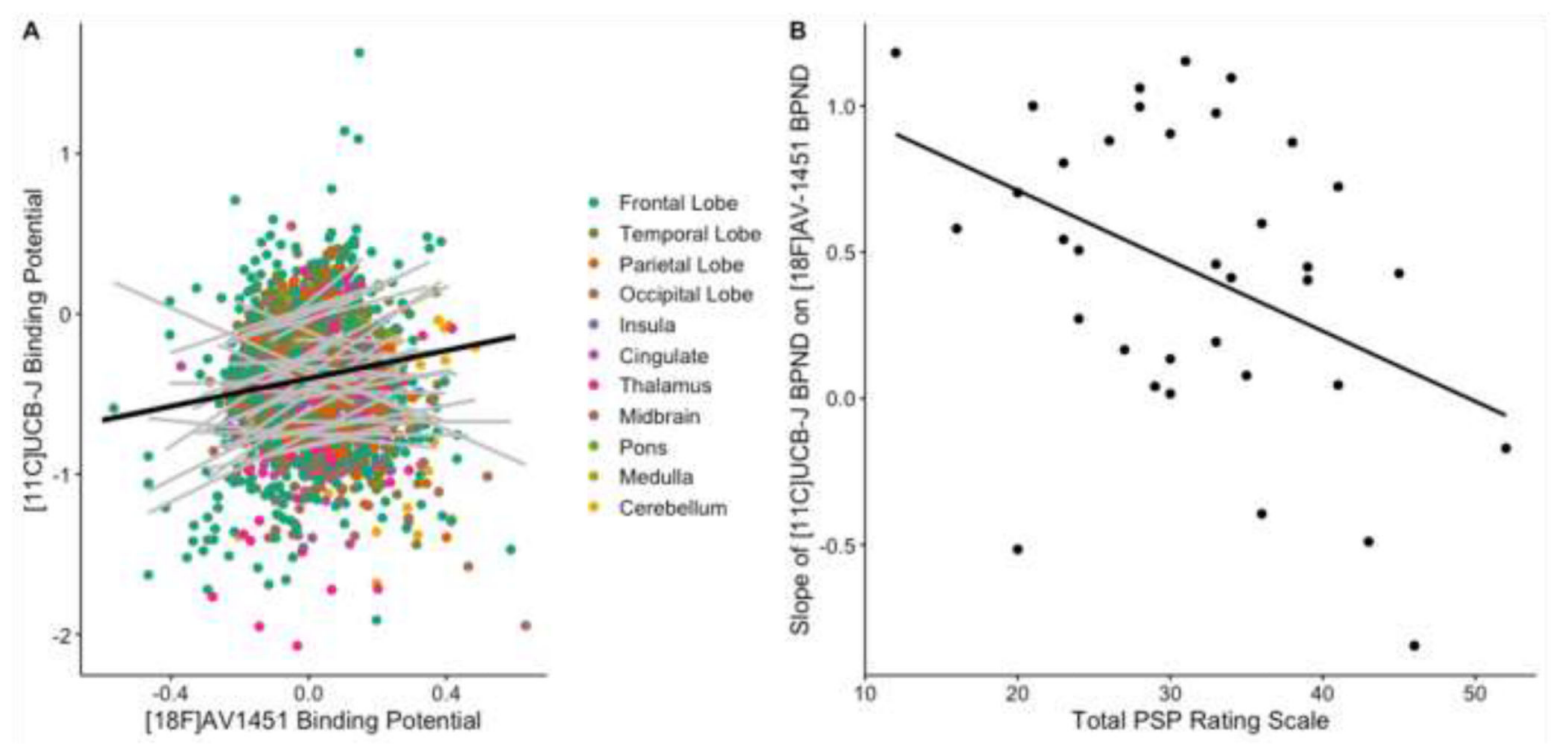
The association between normalised synaptic density ([^11^C]UCB-J) and molecular pathology ([^18^F]AV-1451) is a function of disease severity. A) Scatter plot of [^11^C]UCB-J BP_ND_ and [^18^F]AV-1451 BP_ND_ from 35 patients with PSP-Richardson’s syndrome and amyloid-negative CBS (each grey line represents a patient), across 73 regions of interest (excluding those with previously reported off-target binding, i.e. basal ganglia and substantia nigra) normalised against controls; the dark black line in A depicts the overall fit of the linear mixed model, whilst grey lines represent individual patient data. B) The slope for each individual (i.e. each grey line in A) is negatively correlated with disease severity (as measured with the PSP rating scale); R = −0.41, p < 0.007.

There was an overall positive relationship between normalised [^18^F]AV-1451 BP_ND_ and [^11^C]UCB-J BP_ND_ across the patient cohort (*β=0.4, t=3.6, p=0.001*) (Figure 2A). There was a significant region-by-[^18^F]AV-1451 interaction (*p<0.001*) driven by subregions of the frontal, parietal, and temporal cortices, as well as the hippocampus, subcallosal area, and the thalamus, with all but the hippocampus surviving correction for multiple comparison. Age (p = 0.9) and scan interval (p = 0.5) did not have a significant effect on the overall model (NB: brain regions with known off-target binding of [^18^F]AV-1451 were removed before running this linear mixed model). The direction of the relationship between [^18^F]AV-1451 BP_ND_ and [^11^C]UCB-J BP_ND_ within each individual (i.e. the slope of each grey line in Figure 2A) negatively correlated with disease severity (*β = − 0.02, t = −2.9, p = 0.007, R = −0.41*), independent of age (effect of age: *β=0.02, t=2.6, p= 0.01*) (Figure 2B). In other words, those patients with more severe disease displayed a less positive relationship between [^18^F]AV- 1451 BP_ND_ and [^11^C]UCB-J BP_ND_.

Of note, the positive correlation between [^18^F]AV-1451 BP_ND_ and [^11^C]UCB-J BP_ND_ across the patient cohort remains even if BP_ND_ values are not normalised against the control data (*β=0.4, t=4.0, p=0.0001*), as well as the negative relationship between disease severity and the slope of each individual in Figure 2A. Also note that very similar findings were observed using BP_ND_ derived from data without partial volume correction (Supplementary Figure 2).

The relationship between [^18^F]AV-1451 and [^11^C]UCB-J binding was also positive in an analogous linear mixed effect model in controls alone (*β=0.6, t=4, p<0.0001*), with no main effect of age or scan interval, or [^18^F]AV-1451-by-Region interaction. The relationship between unadjusted [^18^F]AV-1451 and [^11^C]UCB-J binding in all three groups (controls, amyloid negative CBS and PSP) is shown in Supplementary Figure 3.

### Cross-regional correlation between [^18^F]AV-1451 BP_ND_ and [^11^C]UCB-J BP_ND_

**Figure 3:**
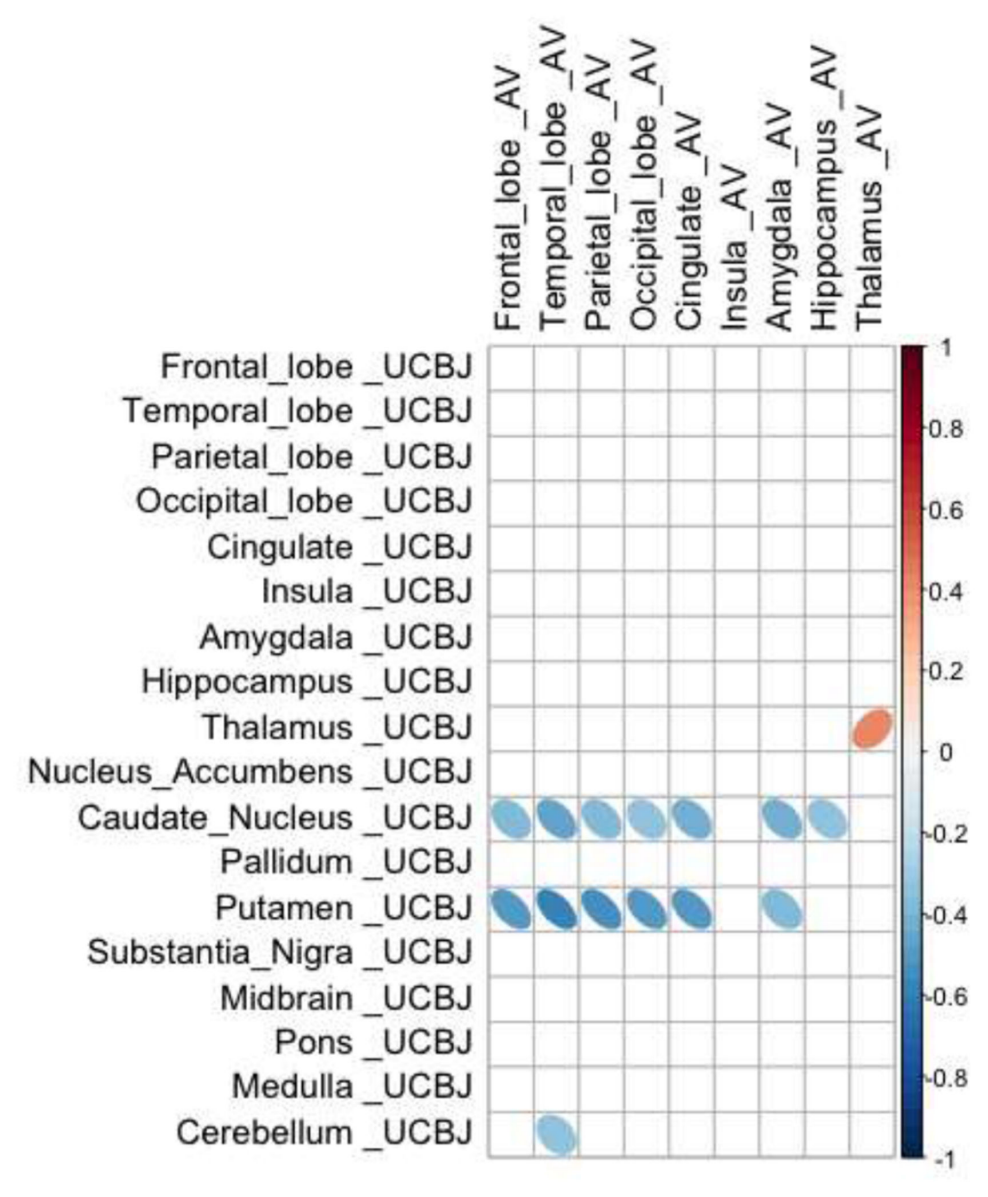
Cortical pathology is negatively correlated with subcortical synaptic density. Correlation, in patients, between normalised [^18^F]AV-1451 BP_ND_ in cortical regions (horizontal axis) and normalised [^11^C]UCB-J BP_ND_ in cortical and subcortical target regions (vertical axis). Negative correlations are observed between cortical [^18^F]AV-1451 BP_ND_ (in frontal, temporal, parietal and occipital cortices), and [^11^C]UCB-J BP_ND_ in the caudate nucleus, putamen and cerebellum. Only significant correlations (at p < 0.05 uncorrected for multiple comparisons) are shown.

Synaptic density in a region is proposed to be affected by both local tau pathology and tau burden in connected regions from which it receives afferent projections. As a result, despite a positive correlation at a regional level, the synaptic density in any given region may be negatively affected by remote insult, with diaschisis between anatomically connected regions (illustrated schematically in Figure 1A). As an exploratory analysis, we computed the asymmetric Pearson’s correlation matrix shown in Figure 3, between normalised cortical [^18^F]AV-1451 BP_ND_ (horizontal axis of matrix) and normalised cortical and subcortical [^11^C]UCB-J BP_ND_ (vertical axis of matrix) in patients. We show that overall, there are significant negative correlations between cortical (frontal, temporal, parietal, occipital) [^18^F]AV-1451 BP_ND_ and subcortical [^11^C]UCB-J BP_ND_ within the caudate nucleus and putamen (-0.52 < R < -0.37; p < 0.05; uncorrected for multiple comparisons). We observed a positive correlation between [^18^F]AV-1451 BP_ND_ and [^11^C]UCB-J BP_ND_ within the thalamus where strong local connections exist (Figure 3). We did not include subcortical [^18^F]AV-1451 BP_ND_ in the matrix in Figure 3 given the off-target binding in these regions which undermines the interpretability of the signal. However, we include these regions as well as other subregions in the larger correlation matrix in Supplementary Figure 4 for completeness. Similar findings are seen using BP_ND_ from data without partial volume correction (Supplementary Figure 5).

## Discussion

We have identified an *in vivo* relationship between molecular pathology (estimated with [^18^F]AV-1451 PET) and synaptic density (estimated with [^11^C]UCB-J PET), in patients with the primary tauopathies of Progressive Supranuclear Palsy and Corticobasal Degeneration (inferred *in vivo* from amyloid-negative corticobasal syndrome). There are three principal results: (i) regions with higher synaptic density have higher molecular pathology, (ii) within regions, synaptic density becomes less dependent on [^18^F]AV-1451 binding as disease severity increases, and (iii) between regions, increased cortical [^18^F]AV-1451 binding is associated with reduced subcortical synaptic density. We interpret these three findings in the context of synaptic connectivity-based susceptibility to tauopathy, the synaptotoxic effects of tauopathy, and cortico-subcortical diaschisis, respectively. The above results are congruent with the model of tau-induced synaptic toxicity, acknowledging the caveat of off-target binding of [^18^F]AV-1451. Our primary pathology of interest in the context of PSP and CBD is 4R-tau, but other tauopathies such as latent Alzheimer pathology in older adults, and non-tau molecular pathologies may also contribute to [^18^F]AV-1451 binding.

The effect of hyperphosphorylated tau on synaptic function and density is complex. It involves both direct and indirect pathways of injury with changes in cellular physiology preceding the loss of neurons. Through direct pathways, pathological tau interferes with dendritic morphology, synaptic protein expression, the number of NMDA (N-methyl-D-Aspartate) and AMPA (α-amino-3-hydroxy-5-methyl-4-isoxazolepropionic acid) receptors on the pre-synaptic membrane, mitochondrial function, synaptic vesicle numbers, and ultimately synaptic loss (for a review of animal studies illustrating various direct tau-induced synaptic abnormalities see Jadhav *et* al. 2015 ^52^). Tau also directly affects the axon cytoskeleton and trafficking, as well as the normal functioning of the soma ^53^. Indirectly, hyperphosphorylated tau adversely affects the functioning of the neuronal support network, including glia cells and astrocytes ^54–56^. These events are affected by the stage and severity of the disease process, and in relation to regional differences in connectivity which we discuss next (concepts schematically illustrated in Figure 1).

We identified a positive relationship between the binding of [^11^C]UCB-J and [^18^F]AV-1451 such that areas of the brain with higher synaptic density had higher pathology. This accords with preclinical and clinical models of tauopathy in which the strength of local network connectivity facilitates the transneuronal spread of tau pathology ^9, 12, 57–59^.

However, the relationship between tau accumulation and synaptic density changes with disease progression, at least as inferred from the cross-sectional moderation by disease severity (Figure 2B). With increasing scores on the PSP rating scale, synaptic density becomes less dependent on local accumulation of pathology. In other words, according to the model (see Figure 1) in areas with relatively low tau accumulation synaptic density is minimally affected, whereas in areas with higher tau accumulation there is reduction of synaptic density as the disease progresses; and this preferentially occurs in synapse rich areas. As the disease progresses, other pathological processes may contribute to synaptic loss, such as inflammation, another predictor of prognosis and mediator of synaptic loss ^60^. There is therefore not a simple linear relationship between tau accumulation and synaptic density in moderate and advanced disease. This observation accords with human post-mortem and animal studies. In post-mortem studies of the tauopathy Alzheimer’s disease, there is a biphasic synaptic protein response during disease progression, with increases in synaptophysin/syntaxin/SNAP-25 in early Braak stages and synaptic loss observed only when the disease has progressed to the neocortex ^19^. In the P301L transgenic mouse model of PSP-like tauopathy, there is a differential loss of synapses, as well as synaptic proteins, depending on disease stage ^20^. These results have recently been replicated *in vivo*, where the relationship between synaptic density and tau burden in patients with Alzheimer’s disease is reported to be modulated by cortical tau load. Coomans *et al*. show that in patients with mild disease and low cortical tau burden, the relationship between tau and synaptic density is positive, whereas in those with increasing cortical tau load, this relationship changes direction ^22^; the relationship between the two tracers in controls is not reported.

In our study, we also observe a positive relationship between [^18^F]AV-1451 and [^11^C]UCB-J binding potentials in controls (Supplementary Figure 3), even though [^18^F]AV-1451 binding is lower in controls. Disease-related [^18^F]AV-1451 binding attributable to presence of PSP/CBD pathology is unlikely in the controls, as the prevalence of these conditions in the normal population is only 1/10,000 ^61^. However, the presence of asymptomatic Alzheimer’s disease pathology in the normal older population is more likley. Rising from the age of 40, by the age of 85 two-thirds of cognitively normal individuals will show positive changes in the A/T/N classification for Alzheimer’s disease, whether by CSF, plasma or amyloid PET ^62, 63^. Some of the non-specific [^18^F]AV-1451 signal, even in healthy controls, may therefore be attributable to latent/pre-clinical AD pathology. We control for this component of the signal by subtracting the mean regional control values from those of the patients.

The positive correlation between [^18^F]AV-1451 and [^11^C]UCB-J binding potential in controls appears stronger compared to that seen in patients, as a group (Supplementary Figure 3). One explanation for this observation is the heterogeneity in disease severity in PSP/CBD, given the interaction between [^11^C]UCB-J, [^18^F]AV-1451 and disease severity. This can be understood in terms of the model set out in Figure 1. The patient group includes those with a strong positive correlation (at early stages of disease) and those with negligible correlation (as a consequence of more advanced disease). The net result for a group-wise test will be a reduction of the group-correlation. This is not present in the control group, in whom the level of tau pathology is expected to be very much lower (even if present from Alzheimer type tau with high [^18^F]AV-1451 affinity).

To understand the biphasic relationship between molecular pathology and synaptic density, one must consider other key players in synaptotoxicity in tauopathies, such as neuroinflammation ^64^. Recent *in vivo* studies have confirmed the regional co-localisation of inflammation and [^18^F]AV-1451 binding in PSP, including in many cortical areas ^65^, in line with previous *in vivo* ^66, 67^, and post-mortem ^68^ reports of the tight interplay between neuroinflammation and tau accumulation in tauopathies. There is growing evidence that these two pathological processes affect synaptic function both independently and synergistically.

The relationship between tauopathy and synaptic density is even more intriguing when considering the change in synaptic density in one region as a function of pathology in another. There are strong correlations between [^11^C]UCB-J binding within the basal ganglia (in particular the caudate nucleus and putamen) and [^18^F]AV-1451 binding in all major cortical areas. The reverse association, between subcortical [^18^F]AV-1451 and cortical [^11^C]UCB-J binding is also observed (Supplementary Figure 4 and 5) but is dismissed here as uninterpretable in view of subcortical off-target binding of [^18^F]AV-1451. The significant negative correlation between cortical [^18^F]AV-1451 binding and synaptic density in the basal ganglia could be a reflection of severe disease in the basal ganglia and accumulating pathology in the neocortex. In other words, synapses are severely affected in the basal ganglia as one of the earliest sites of pathology, with pathology spreading and accumulating in synapse-rich areas of the brain, for example the neocortex. A second possible explanation is that loss of descending cortico-striatal axons due to cortical pathology, may cause diaschisis, affecting subcortical synaptic density even further. Previous analysis of diffusion tensor imaging in patients with PSP/CBD have revealed extensive white matter abnormalities (within the main association fibres) beyond the degree of cortical atrophy ^69, 70^ resulting in loss of cortical afferents onto subcortical structures. A third, though not mutually exclusive, potential explanation is the weakening of cortical-subcortical functional connectivity resulting from dysfunctional synapses rather than synaptic loss, although cortico-subcortical connectivity is inferred and was not directly measured in our study.

Although at a regional level there is a positive correlation between [^11^C]UCB-J and [^18^F]AV-1451 BP_ND_, we are not directly measuring either synaptic function or the synaptotoxic tau oligomers. This caveat must be borne in mind when interpreting PET data. It is the preclinical models that have shown that oligomers of tau are toxic to synaptic function, even in the absence of tau polymers/fibrils ^15, 16^. By the time tau aggregates are established, oligomers of tau are expected cortically, and perhaps interfering with cortical function and the integrity of descending axons.

There are other limitations to our study. First, the low affinity of [^18^F]AV-1451 for PSP and CBD 4R tau. Even though the radioligand recapitulates the distribution of post-mortem neuropathology in PSP and CBD, and binds PSP 4R tau, the affinity is very much lower than for 3R tau in Alzheimer’s disease. Second, there is well-established off-target binding of [^18^F]AV-1451, particularly within subcortical structures where monoamine oxidase and neuromelanin are present. Off-target binding is most prominent in the basal ganglia and substantia nigra which we excluded before running the linear mixed model and correlation matrix. We included these regions in the detailed descriptive correlation matrices in Supplementary Figure 4 and 5 for completeness sake, noting the strong negative correlations between cortical [^18^F]AV-1451 BP_ND_ and subcortical [^11^C]UCB-J BP_ND_. Furthermore, we normalised our patient data against that of controls to remove any additional normal levels of off-target binding, noting the caveat that the remaining signal in patients may still arise from tau and non-tau pathology; there is no evidence to suggest that [^18^F]AV-1451 shares a common binding target with [^11^C]UCB-J, which has a high specificity for SV2A in previous *in vivo* and *in vitro* validation studies ^38, 71^. Third, we note that in PET studies of neurodegeneration with atrophy, grey matter volume loss can affect the interpretation of PET signals. However, synaptic loss in PSP and CBD occurs even in areas of the brain without discernible atrophy on MRI ^7, 8^. Nonetheless, we used a stringent partial volume correction method (GTM) to minimise the effect of atrophy on our ligand cross-correlations. Of note, our data without partial volume correction yield similar results in all the main analyses (Supplementary Figures 2 and 5). Fourth, although the sample size is small, it is adequately powered in view of the large effect sizes seen. However, subtler relationships with phenotypic variants of PSP and CBS, would require larger studies. Additionally, clinical diagnostic criteria for PSP-Richardson’s syndrome and amyloid negative CBS (here called CBD) were used to select a clinical cohort with likely a 4R-tauopathy as the underlying pathological diagnosis. Whilst both PSP-Richardson’s syndrome and amyloid negative corticobosal syndrome are highly correlated with a 4R-tauopathy at post-mortem, both from our local brain bank and internationally ^26, 27, 72^, other pathologies are possible, and so are coexistent pathologies that may synergistically contribute to neurodegeneration ^73^. Neuropathological correlates, to test the correlations between phenotype and pathology, and between *in vivo-*to-post-mortem measures of synaptic density, as well as tau-to-synapse correlations would be useful but are not yet available for our cohort. Lastly, the cross-sectional design of this study limits the interpretation of the dynamic relationship between pathology and synaptic loss. Although we include patients at various stages of their illness, a longitudinal design is necessary to test the dynamic relationship we propose, and the mediation of synaptic loss by progressive tauopathy.

In conclusion, we demonstrate a widespread positive association between [^18^F]AV-1451 and [^11^C]UCB-J binding in patients with symptomatic PSP and amyloid-negative corticobasal syndromes. Individual variability in this association correlates with disease severity. The complex relationship between molecular pathology, including but not exclusive to tau, and synaptic density may explain changes in cognitive and motor physiology. We hope that these insights will inform the design of new clinical trials to arrest PSP and CBD.

## Supplementary Material

Supplementary Material

## Figures and Tables

**Table 1:** Clinical and Demographics summary. Results are given as mean (and standard deviation) unless otherwise stated. PSP refers to patients with PSP-Richardson’s syndrome. CBD refers to amyloid negative corticbasal syndrome. The F-statistic and p-values are derived from ANOVA. ACE-R: revised Addenbrooke’s Cognitive Examination, MMSE: Mini-mental State Examination, PSPRS: Progressive Supranuclear Palsy Rating Scale, CBFS: Cortical Basal ganglia Functional Scale, INECO: Institute of Cognitive Neurology frontal screening tool. ^a^ chi-squared test. ns = non-significant at *p<0.05*.

	Control	PSP	cbd	F (*p*)
**Gender (M:F)**	11:8	10:13	7:5	*ns^a^*
**Age at [^11^C]UCB-J PET in years**	68.9 (7.1)	71.3 (8.6)	70.9 (7.9)	*ns*
**Symptom duration (years)**	-	3.9 (2.2)	3.9 (2.1)	*ns*
**Education (years)**	13.6 (2.8)	12 (4.2)	12.6 (2.8)	*ns*
**ACE-R total (max. 100)**	96.7 (2.7)	80.9 (12.4)	77.5 (17.1)	10.1 (*<0.001*)
Attention Orientation (max .18)	17.9 (0.3)	16.3 (1.9)	16.3 (2.3)	4.5 (*0.02*)
Memory (max .26)	24.6 (1.7)	21.8 (3.8)	20.9 (5.3)	5.3 (*0.01)*)
Fluency (max . 14)	12.8 (1.0)	6.6 (3)	7.2 (3.5)	28.0 (*<0.001*)
Language (max .26)	25.6 (0.8)	23.3 (4.2)	21 (7.2)	5.4 (*0.01*)
Visuospatial (max .16)	15.7 (0.6)	12.8 (3.4)	12.1 (4.6)	7.5 (*0.001*)
**MMSE (max. 30)**	29.4 (1.2)	26.9 (2.6)	25.3 (4.9)	6.7 (*0.002*)
**INECO (max. 30)**	25.7 (2.1)	17.2 (5.4)	15.4 (6.5)	17.9 (*<0.0001*)
**PSPRS (max. 100)**	-	32.7 (8.2)	28.9 (10.0)	3.4 (*0.07*)
**CBFS (max. 120)**	-	32.7 (15.9)	26.2 (16.2)	0.2 (0.7)
**Injected activity (MBq)**				
**[^11^C]UCB-J**	370.7 (114.3)	322.2 (86.0)	320.4 (113.8)	*ns*
**[^18^F]AV-1451**	182.3 (10.8)	182.1 (11.4)	186.1 (11.1)	*ns*
**[^11^C]UCB-J and [^18^F]AV-1451**	157.2 (125.6)	155.9 (129.2)	45.5 (65.7)	4.6 (*0.02*)
**PET scan interval (in days)**				

## Abbreviations

PSP: Progressive Supranuclear Palsy – Richardson’s Syndrome
CBD: Corticobasal Degeneration
CBS: Corticobasal Syndrome
PSPRS: PSP Rating Scale
