# Supplementary material for: Molecular pathology and synaptic loss in primary tauopathies: A [18F]AV-1451 and [11C]UCB-J PET study
Source: Brain. Author manuscript; Available in PMC 2022 Mar 31. (PMC8967099; doi:10.1093/brain/awab282)
Supplement: Supplementary Material [file EMS133107-supplement-Supplementary_Material.pdf]

Molecular pathology and synaptic loss in primary tauopathies: A [^{18}F]AV-1451 and [^{11}C]UCB-J PET study

Supplementary Figures = 5
Supplementary Tables = 4

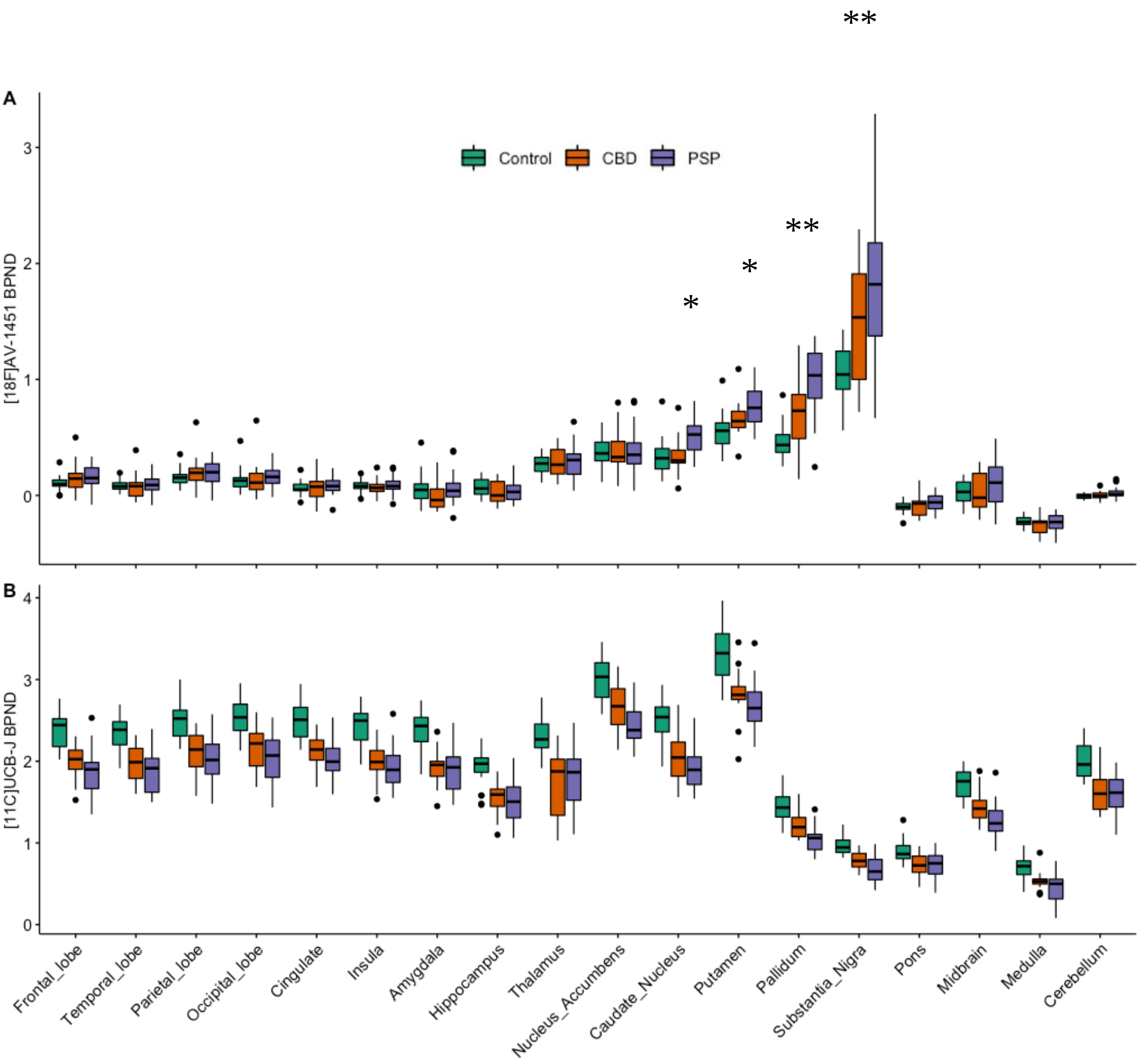

Supplementary Figure 1. Boxplots of regional [^{18}F]AV-1451 (A) and [^{11}C]UCB-J (B) non-displaceable binding potentials (BP_{ND}) across 18 regions of interest formed by aggregating Hammersmith atlas regions. Significantly higher [^{18}F]AV-1451 binding potentials are seen in patients within the basal ganglia, and substantia nigra compared to controls (depicted with a black star). Patients with PSP and amyloid-negative CBS (CBD) have lower synaptic density in all the 18 regions illustrated in B, $p<0.05$ (FDR corrected).

	Control (N = 19)	CBD (N = 12)	PSP (N = 23)
**Frontal Lobe**			
min	0	-0.045	-0.08
max	0.285	0.5	0.335
median (IQR)	0.10 (0.08, 0.13)	0.16 (0.10, 0.19)	0.15 (0.09, 0.25)
**Parietal Lobe**			
min	0.01	-0.06	-0.085
max	0.195	0.39	0.27
median (IQR)	0.08 (0.04, 0.11)	0.08 (-0.01, 0.12)	0.09 (0.05, 0.14)
**Temporal Lobe**			
min	0.04	-0.02	-0.045
max	0.355	0.63	0.375
median (IQR)	0.15 (0.09, 0.18)	0.20 (0.15, 0.25)	0.20 (0.11, 0.28)
**Occipital Lobe**			
min	0.005	-0.035	-0.015
max	0.47	0.645	0.365
median (IQR)	0.12 (0.07, 0.15)	0.12 (0.05, 0.20)	0.18 (0.09, 0.22)
**Cingulate**			
min	-0.06	-0.14	-0.125
max	0.22	0.315	0.235
median (IQR)	0.05 (0.03, 0.09)	0.08 (0.02, 0.13)	0.08 (0.04, 0.14)
**Insula**			
min	-0.03	-0.02	-0.075
max	0.19	0.24	0.24
median (IQR)	0.08 (0.05, 0.11)	0.07 (0.04, 0.11)	0.07 (0.06, 0.13)
**Amygdala**			
min	-0.135	-0.12	-0.195
max	0.455	0.285	0.385
median (IQR)	0.05 (-0.03, 0.12)	-0.03 (-0.09, 0.07)	0.04 (-0.03, 0.11)
**Hippocampus**			
min	-0.055	-0.115	-0.095
max	0.2	0.185	0.26
median (IQR)	0.03 (0.00, 0.15)	0.04 (-0.05, 0.12)	0.02 (-0.04, 0.11)
**Thalamus**			
min	0.11	0.095	0.04
max	0.405	0.495	0.635
median (IQR)	0.27 (0.21, 0.33)	0.28 (0.18, 0.40)	0.31 (0.18, 0.38)
**Nucleus Accumbens**			
min	0.115	0.08	0.04
max	0.63	0.8	0.815
median (IQR)	0.34 (0.30, 0.43)	0.35 (0.30, 0.47)	0.36 (0.26, 0.45)
**Caudate Nucleus**			

min	0.12	0.06	0.255
max	0.81	0.755	0.815
median (IQR)	0.30 (0.21, 0.38)	0.33 (0.28, 0.39)	0.53 (0.42, 0.62)
**Pallidum**			
min	0.25	0.38	0.245
max	0.865	1.295	1.375
median (IQR)	0.42 (0.37, 0.52)	0.76 (0.56, 0.92)	1.07 (0.86, 1.23)
**Putamen**			
min	0.295	0.55	0.485
max	0.99	1.09	1.105
median (IQR)	0.52 (0.43, 0.61)	0.65 (0.60, 0.73)	0.76 (0.65, 0.91)
**Substantia Nigra**			
min	0.56	0.8	0.665
max	1.43	2.295	3.29
median (IQR)	1.00 (0.91, 1.24)	1.57 (1.26, 1.96)	1.85 (1.38, 2.24)
**Midbrain**			
min	-0.16	-0.21	-0.25
max	0.18	0.29	0.49
median (IQR)	0.03 (-0.04, 0.10)	0.03 (-0.12, 0.20)	0.12 (-0.06, 0.26)
**Pons**			
min	-0.24	-0.22	-0.2
max	-0.04	0.13	0.07
median (IQR)	-0.10 (-0.12, -0.07)	-0.07 (-0.15, -0.04)	-0.06 (-0.11, -0.01)
**Medulla**			
min	-0.31	-0.4	-0.4
max	-0.14	-0.1	-0.12
median (IQR)	-0.23 (-0.26, -0.20)	-0.25 (-0.33, -0.22)	-0.23 (-0.28, -0.17)
**Cerebellum**			
min	-0.045	-0.065	-0.055
max	0.02	0.085	0.14
median (IQR)	-0.01 (-0.03, 0.01)	-0.01 (-0.03, 0.02)	0.01 (-0.00, 0.04)

Supplementary Table 1. Summary statistics for regional [^{18}F]AV-1451 non-displaceable binding potentials in controls, PSP, and amyloid negative CBS (CBD). min = minimum, max = maximum, IQR = interquartile range.

**Contrast**	**Region**	**Estimate**	**SE**	**t-ratio**	**p-value**	**Adjusted p-value (FDR)**
Control - PSP	Caudate Nucleus	-0.188889	0.05028779	-3.75616	0.00056	0.00378
Control - PSP	Pallidum	-0.5344782	0.05028779	-10.628389	0.00000	0.00000
Control - PSP	Putamen	-0.2039648	0.05028779	-4.05595	0.00017	0.00152
Control - PSP	Substantia Nigra	-0.7293099	0.05028779	-14.502722	0.00000	0.00000
Control – CBD	Pallidum	-0.2392405	0.06114926	-3.912402	0.00030	0.00234
Control - CBD	Substantia Nigra	-0.4502545	0.06114926	-7.363204	0.00000	0.00000
CBD - PSP	Caudate Nucleus	-0.1805226	0.05911996	-3.053497	0.00670	0.04020
CBSD- PSP	Pallidum	-0.2952377	0.05911996	-4.993875	0.00000	0.00003
CBD - PSP	Substantia Nigra	-0.2790554	0.05911996	-4.720155	0.00001	0.00010

Supplementary Table 2. Significant regional differences in [^{18}F]AV-1451 non-displaceable binding potential between patients with PSP and amyloid-negative CBS (CBD) compared to controls (corrected for multiple comparison across 18 regions of interest - including combined left and right cortical and subcortical areas of the modified Hammersmith Atlas).

	Control (N = 19)	CBD (N = 12)	PSP (N = 23)
**Frontal Lobe**			
min	2.52	2.025	2.06
max	3.245	2.885	3.325
median (IQR)	2.96 (2.70, 3.06)	2.59 (2.50, 2.80)	2.37 (2.23, 2.58)
**Temporal Lobe**			
min	2.135	1.96	1.755
max	2.96	2.6	2.675
median (IQR)	2.67 (2.45, 2.77)	2.25 (2.09, 2.40)	2.17 (1.92, 2.34)
**Parietal Lobe**			
min	2.61	2.255	2.135
max	3.465	3.18	3.425
median (IQR)	3.12 (2.87, 3.19)	2.63 (2.52, 2.83)	2.52 (2.38, 2.81)
**Occipital Lobe**			
min	2.455	2.25	1.82
max	3.46	3.005	3.22
median (IQR)	2.91 (2.69, 3.09)	2.62 (2.43, 2.71)	2.48 (2.20, 2.73)
**Cingulate**			
min	2.52	2.085	2.03
max	3.425	2.995	3.02
median (IQR)	3.01 (2.81, 3.11)	2.57 (2.54, 2.78)	2.38 (2.25, 2.62)
**Insula**			
min	2.11	1.765	1.78
max	3.03	2.7	2.82
median (IQR)	2.75 (2.49, 2.83)	2.19 (2.09, 2.35)	2.06 (1.89, 2.33)
**Amygdala**			
min	1.875	1.565	1.5
max	3.035	2.535	2.74
median (IQR)	2.60 (2.36, 2.78)	2.11 (1.96, 2.18)	2.06 (1.85, 2.31)
**Hippocampus**			
min	1.265	1.05	1.025
max	2.335	1.805	2.15
median (IQR)	1.86 (1.78, 2.06)	1.58 (1.44, 1.68)	1.44 (1.31, 1.74)
**Thalamus**			
min	2.21	1.005	1.22
max	3.375	2.915	2.96
median (IQR)	2.77 (2.58, 2.96)	2.19 (1.55, 2.45)	2.29 (1.85, 2.40)
**Nucleus Accumbens**			
min	3.315	2.9	2.61
max	4.565	4.39	4
median (IQR)	3.90 (3.65, 4.10)	3.65 (3.47, 3.88)	3.16 (3.07, 3.52)
**Caudate Nucleus**			

min	2.235	1.92	1.82
max	3.515	3.305	3.11
median (IQR)	3.06 (2.82, 3.26)	2.50 (2.23, 2.75)	2.41 (2.16, 2.50)
**Pallidum**			
min	1.415	1.325	0.815
max	2.425	2.125	1.8
median (IQR)	1.80 (1.70, 1.94)	1.62 (1.40, 1.72)	1.24 (1.04, 1.35)
**Putamen**			
min	3.195	2.33	2.61
max	4.72	4.075	4.055
median (IQR)	3.88 (3.66, 4.18)	3.36 (3.31, 3.53)	3.17 (2.93, 3.35)
**Substantia Nigra**			
min	1.71	1.125	0.52
max	2.67	2.175	2.335
median (IQR)	2.07 (1.92, 2.37)	1.78 (1.48, 2.00)	1.53 (0.92, 1.84)
**Midbrain**			
min	1.66	1.25	0.69
max	2.83	2.41	2.55
median (IQR)	2.29 (2.10, 2.45)	1.61 (1.45, 2.10)	1.35 (0.99, 1.61)
**Pons**			
min	0.75	0.45	0.39
max	1.36	1.01	1.06
median (IQR)	0.94 (0.84, 1.02)	0.74 (0.65, 0.88)	0.76 (0.62, 0.90)
**Medulla**			
min	0.33	0.25	-0.05
max	0.95	0.83	0.74
median (IQR)	0.67 (0.58, 0.72)	0.46 (0.39, 0.48)	0.41 (0.21, 0.53)
**Cerebellum**			
min	1.805	1.36	1.19
max	2.535	2.3	2.115
median (IQR)	2.07 (1.93, 2.30)	1.71 (1.53, 1.87)	1.74 (1.54, 1.89)

Supplementary Table 3. Summary statistics for regional [^{11}C]UCB-J non-displaceable binding potentials in controls, PSP, and amyloid negative CBS (CBD). min = minimum, max = maximum, IQR = interquartile range.

Contrast	Region	Estimate	SE	t-ratio	p-value	Adjusted p-value (FDR)
Control - PSP	Amygdala	0.43	0.10	4.42	0.0001	0.0002
Control - PSP	Caudate Nucleus	0.65	0.10	6.64	0.0000	0.0000
Control - PSP	Cerebellum	0.41	0.10	4.21	0.0001	0.0004
Control - PSP	Cingulate	0.52	0.10	5.29	0.0000	0.0000
Control - PSP	Frontal lobe	0.44	0.10	4.54	0.0000	0.0001
Control - PSP	Hippocampus	0.35	0.10	3.55	0.0014	0.0034
Control - PSP	Insula	0.50	0.10	5.11	0.0000	0.0000
Control - PSP	Medulla	0.30	0.10	3.08	0.0068	0.0125
Control - PSP	Midbrain	0.90	0.10	9.24	0.0000	0.0000
Control - PSP	Nucleus Accumbens	0.62	0.10	6.34	0.0000	0.0000
Control - PSP	Occipital lobe	0.44	0.10	4.54	0.0000	0.0001
Control - PSP	Pallidum	0.62	0.10	6.35	0.0000	0.0000
Control - PSP	Parietal lobe	0.42	0.10	4.27	0.0001	0.0003
Control - PSP	Putamen	0.71	0.10	7.30	0.0000	0.0000
Control - PSP	Substantia Nigra	0.73	0.10	7.49	0.0000	0.0000
Control - PSP	Temporal lobe	0.44	0.10	4.49	0.0000	0.0001
Control - PSP	Thalamus	0.59	0.10	6.02	0.0000	0.0000
Control – CBD	Amygdala	0.44	0.12	3.77	0.0007	0.0018
Control – CBD	Caudate Nucleus	0.48	0.12	4.11	0.0002	0.0005
Control – CBD	Cerebellum	0.40	0.12	3.49	0.0018	0.0040
Control – CBD	Cingulate	0.37	0.12	3.19	0.0048	0.0092
Control – CBD	Frontal lobe	0.31	0.12	2.69	0.0215	0.0332
Control – CBD	Hippocampus	0.34	0.12	2.92	0.0111	0.0187
Control – CBD	Insula	0.43	0.12	3.72	0.0008	0.0020
Control – CBD	Midbrain	0.53	0.12	4.53	0.0000	0.0001
Control – CBD	Occipital lobe	0.32	0.12	2.74	0.0186	0.0296
Control – CBD	Parietal lobe	0.36	0.12	3.07	0.0069	0.0125
Control – CBD	Putamen	0.54	0.12	4.61	0.0000	0.0001
Control – CBD	Substantia Nigra	0.42	0.12	3.60	0.0012	0.0030
Control – CBD	Temporal lobe	0.34	0.12	2.92	0.0110	0.0187
Control – CBD	Thalamus	0.72	0.12	6.19	0.0000	0.0000
CBD - PSP	Midbrain	0.38	0.11	3.32	0.0032	0.0066
CBD - PSP	Nucleus Accumbens	0.38	0.11	3.34	0.0029	0.0064
CBD - PSP	Pallidum	0.37	0.11	3.24	0.0041	0.0083
CBD - PSP	Substantia Nigra	0.31	0.11	2.76	0.0174	0.0285

Supplementary Table 4. Significant regional differences in [^{11}C]UCB-J non-displaceable binding potential between patients with PSP and amyloid-negative CBS (CBD) compared to controls, with all surviving correction for multiple comparison across 18 combined regions of interest (aggregated areas of the modified Hammersmith atlas).

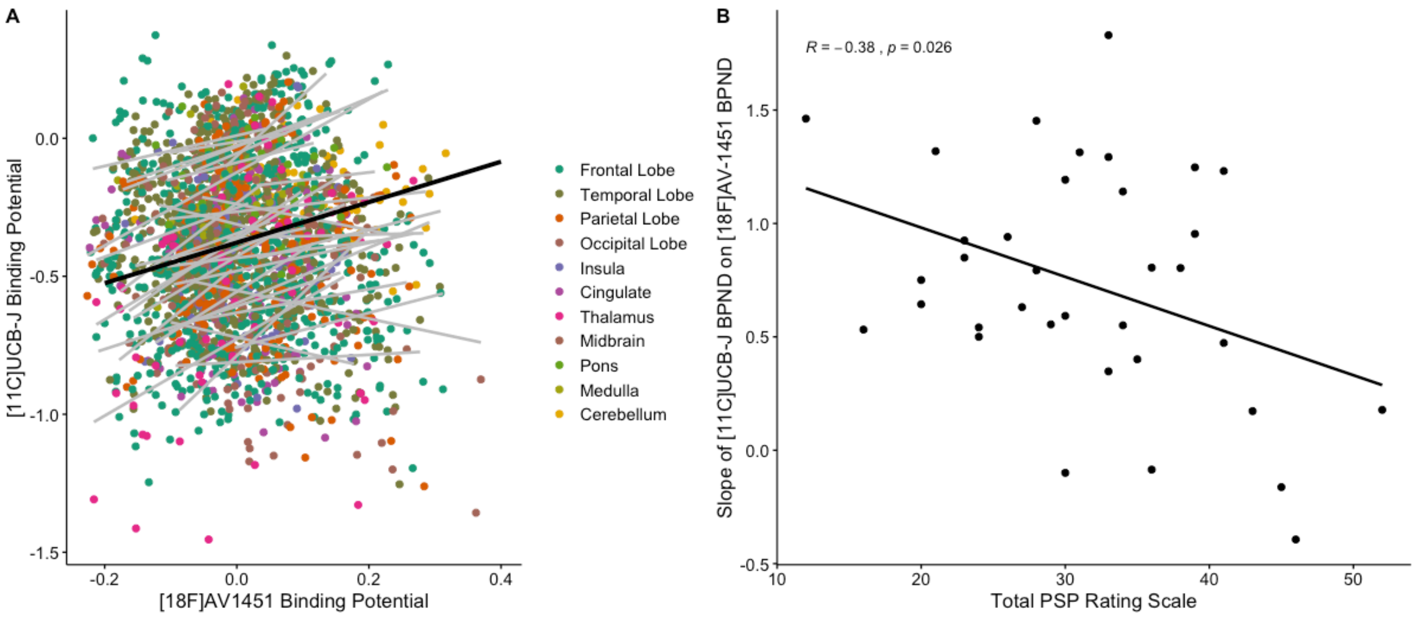

Supplementary Figure 2. The association between synaptic density ([^{11}C]UCB-J) and molecular pathology ([^{18}F]AV-1451) is a function of disease severity. A) Scatter plot of [^{11}C]UCB-J and [^{18}F]AV-1451 non-displaceable binding potential (BP_{ND}) without partial volume correction from 23 patients with PSP and 12 patients with amyloid-negative corticobasal syndrome . Each grey line in A represents data from an individual patient across 73 regions of interest (excluding those with previously reported off-target binding, i.e. basal ganglia and substantia nigra); the black line illustrates the overall model. B) The slope for each individual (i.e. each grey line in A) is negatively correlated with disease severity (as measured with the PSP rating scale); R= -0.38, p = 0.03.

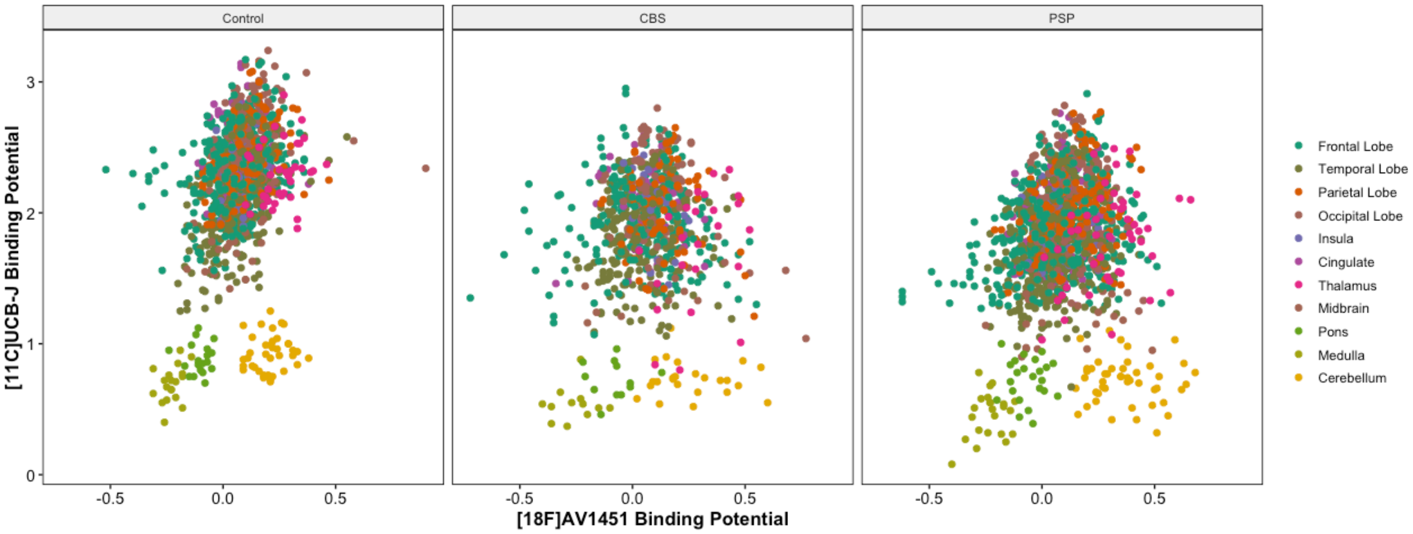

Supplementary Figure 3. Scatterplots of raw [^{18}F]AV-1451 BP_{ND} against [^{11}C]UCB-J BP_{ND}, in controls, and patients with PSP and amyloid negative CBS, for all cortical and subcortical regions except those where there is known off-target binding of [^{18}F]AV-1451.

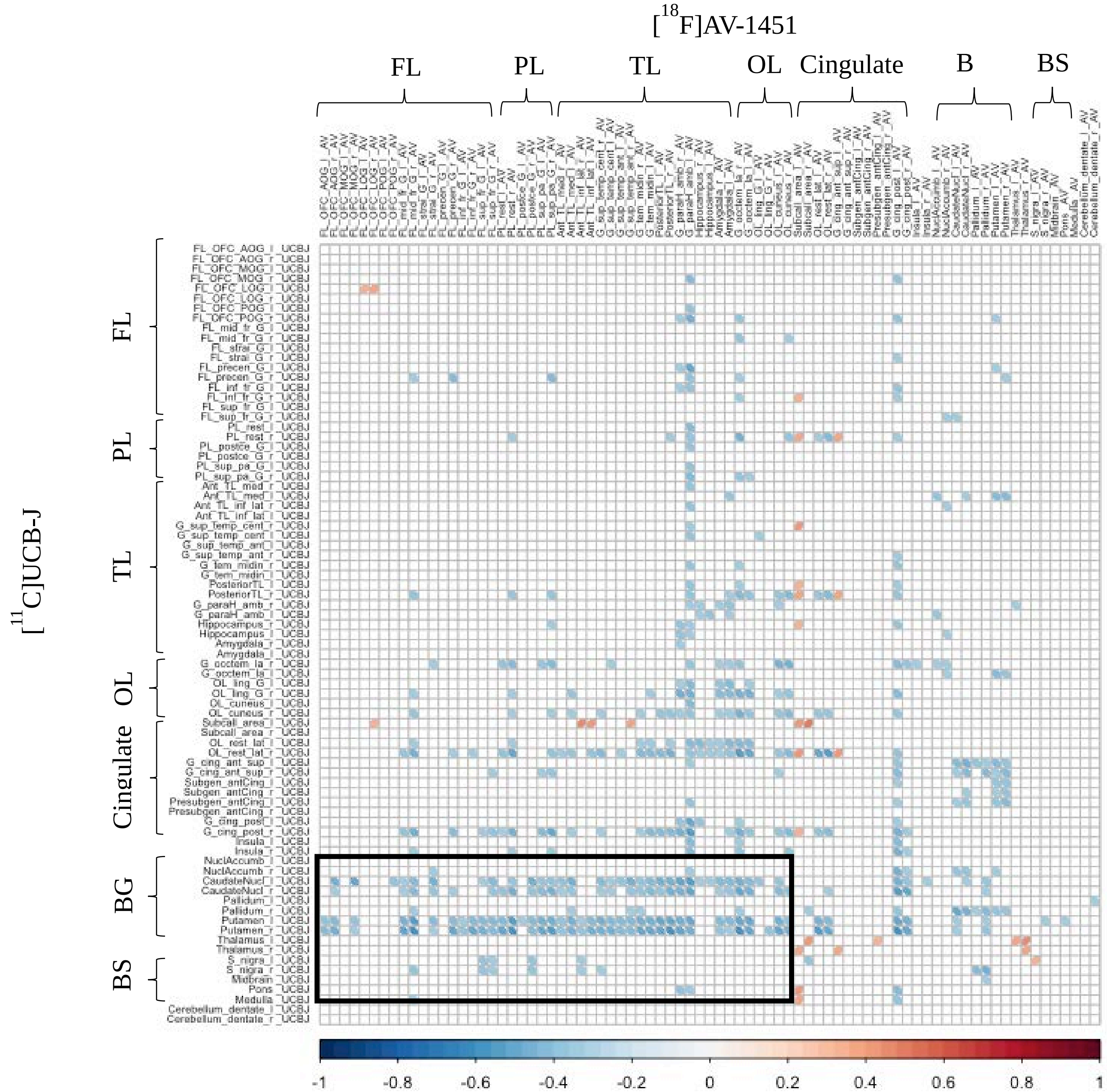

Supplementary Figure 4. Cortical molecular pathology is negatively correlated with subcortical synaptic density (data with partial volume correction). Correlation between [^{18}F]AV-1451 BP_{ND} in a source region (horizontal axis) and [^{11}C]UCB-J BP_{ND} in a target region (vertical axis), across 79 regions of interest in patients, using non-displaceable binding potential (BP_{ND}) determined from data with partial volume correction. The black box in the lower left quadrant focuses on cortical [^{18}F]AV-1451 BP_{ND} and subcortical [^{11}C]UCB-J BP_{ND}. Abbreviations: l: left, r: right, FL: Frontal Lobe, OFC: Orbitofrontal Cortex, AOG: Anterior Orbital Gyrus, MOG: Middle Orbital Gyrus, LOG: Lateral Orbital Gyrus, POG: Posterior Orbital Gyrus, mid_fr_G: Middle Frontal Gyrus, strai_G: Straight Gyrus, precen_G: Precentral Gyrus, inf_fr_G: Inferior Frontal Gyrus, sup_fr_G: Superior Frontal Gyrus; PL: Parietal Lobe, postce_G: Postcentral Gyrus, sup_pa_G: Superior Parietal Gyrus: TL: Temporal Lobe, Ant_med: Anterior Medial, Ant_inf_lat: Anterior Inferiolateral, "G_sup_temp_cent: Superior Temporal Gyrus – superior part, G_sup_temp_ant: Superior Temporal Gyrus- Anterior part, G_tem_midin_r: Middle and Inferior Temporal Gyrus, G_paraH_amb: Parahippocampal and ambient gyri, G_occtem_la: Occipitotemporal Gyrus – Lateral Part (Fusiform Gyrus), OL: Occipital Lobe, OL_ling_G_l: Lingual Gyrus, Subcall_area: Subcallosal Area, OL_rest_lat: Latera Remainder of Occipital Lobe, G_cing_ant_sup: Cingulate Gyrus Anterior Part, Subgen_antCing: Subgenual Frontal Lobe, Presubgen_antCing: Presubgenual Frontal Lobe, G_cing_post: Cingulate Gyrus – posterior part; BG: Basal Ganglia; BS: Brainstem. Significant correlations at $p<0.05$ (FDR uncorrected) are shown in the matrix above.

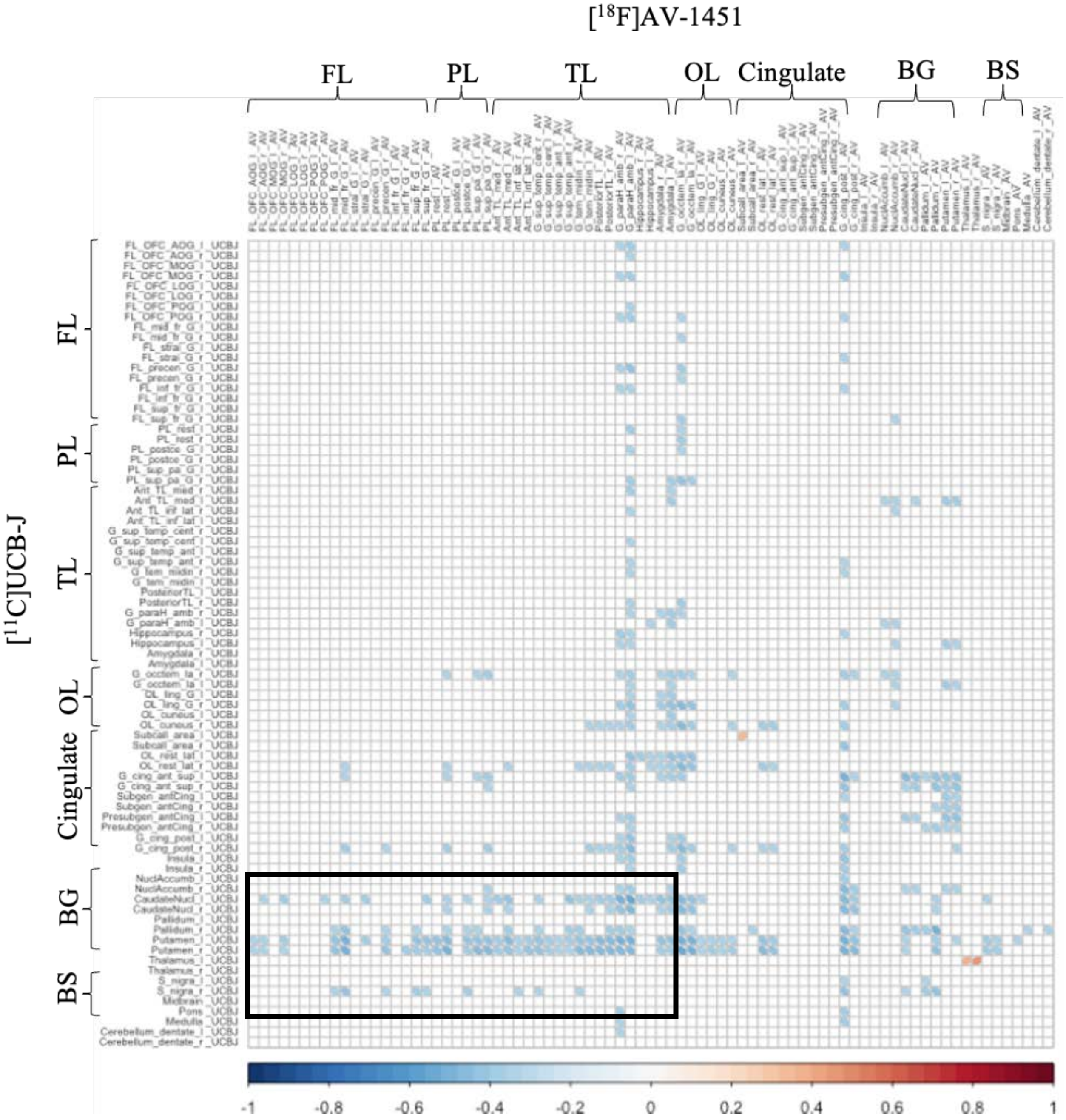

Supplementary Figure 5. Cortical molecular pathology is negatively correlated with subcortical synaptic density (data without partial volume correction). Correlation between [18F]AV-1451 BP_{ND} in a source region (horizontal axis) and [11C]UCB-J BP_{ND} in a target region (vertical axis), across 79 regions of interest in patients, using non-displaceable binding potential (BP_{ND}) determined from data without partial volume correction. The black box in the lower left quadrant focuses on cortical [18F]AV-1451 BP_{ND} and subcortical [11C]UCB-J BP_{ND}. Abbreviations: l: left, r: right, FL: Frontal Lobe, OFC: Orbitofrontal Cortex, AOG: Anterior Orbital Gyrus, MOG: Middle Orbital Gyrus, LOG: Lateral Orbital Gyrus, POG: Posterior Orbital Gyrus, mid_fr_G: Middle Frontal Gyrus, strai_G: Straight Gyrus, precen_G: Precentral Gyrus, inf_fr_G: Inferior Frontal Gyrus, sup_fr_G: Superior Frontal Gyrus; PL: Parietal Lobe, postce_G: Postcentral Gyrus, sup_pa_G: Superior Parietal Gyrus: TL: Temporal Lobe, Ant_med: Anterior Medial, Ant_inf_lat: Anterior Inferiolateral, G_sup_temp_cent: Superior Temporal Gyrus – superior part, G_sup_temp_ant: Superior Temporal Gyrus - Anterior part, G_tem_midin_r: Middle and Inferior Temporal Gyrus, G_paraH_amb: Parahippocampal and ambient gyri, G_occtem_la: Occipitotemporal Gyrus – Lateral Part (Fusiform Gyrus), OL: Occipital Lobe, OL_ling_G_l: Lingual Gyrus, Subcall_area: Subcallosal Area, OL_rest_lat: Lateral Remainder of Occipital Lobe, G_cing_ant_sup: Cingulate Gyrus Anterior Part, Subgen_antCing: Subgenual Frontal Lobe, Presubgen_antCing: Presubgenual Frontal Lobe, G_cing_post: Cingulate Gyrus – posterior part; BG: Basal Ganglia; BS: Brainstem. Significant correlations at $p<0.05$ (FDR uncorrected) are shown in the matrix above.
